# Endodontic management of maxillary premolars with three roots: a case series

**DOI:** 10.3389/froh.2026.1758340

**Published:** 2026-02-24

**Authors:** Ayah Ali, Abdul Rahman Saleh, Firas Elmsmari, Abayomi O. Baruwa

**Affiliations:** 1 Department of Clinical Sciences, College of Dentistry, Ajman University, Ajman, United Arab Emirates; 2 Center of Medical and Bio-allied Health Sciences Research, Ajman University, Ajman, United Arab Emirates

**Keywords:** case series, cone beam computed tomography, maxillary premolars, root anatomy, root canal treatment

## Abstract

**Background:**

Three-rooted maxillary premolars represent rare anatomical variations that pose significant diagnostic and clinical challenges in endodontic treatment. Failure to recognize additional roots or canals increases the risk of persistent infection and treatment failure. This case series describes the diagnostic approach and clinical management of maxillary premolars with complex three-rooted anatomy, emphasizing the importance of advanced imaging and magnification in achieving successful treatment outcomes.

**Case description:**

Three patients were referred for endodontic evaluation of maxillary premolars with suspected complex anatomy. Clinical examination, pulp testing, and periapical radiographs were supplemented with cone beam computed tomography (CBCT). All three cases exhibited distinct three-rooted morphology, with CBCT scans proving essential for identifying bifurcations, confluence, and previously missed canals. Access cavity modification with ultrasonic tips facilitated straight-line access and detection of additional canals. Chemo-mechanical preparation was performed with 2% sodium hypochlorite and 17% ethylenediaminetetraacetic acid using rotary instruments. Following obturation, postoperative radiographs were taken to verify treatment quality.

**Conclusion:**

This case series emphasizes the importance of recognizing anatomical variations in maxillary premolars. It also demonstrates the essential role of CBCT imaging and magnification in the diagnosis and management of complex root canal systems.

## 1 Introduction

The root canal anatomy system is quite complex, with intricacies that support the colonization and multiplication of microbial flora (1). Therefore, the efficacy of any endodontic treatment depends on the identification of all canals, followed by thorough shaping, cleaning, and three-dimensional sealing of the root canal space (2). Limited knowledge of possible variations in root morphology and canal configurations often leads to residual necrotic tissue and bacteria, which increases the risk of treatment failure (3). Indeed, a missed or untreated canal is considered one of the leading causes of failure in endodontics (4). Generally, failed endodontic treatments may show no symptoms or, in some instances, may present with acute symptoms, underscoring the need for meticulous care to avoid complications (5). A previous study on anatomy by Kartal et al., based on the classification by Vertucci, explored the configurations of the maxillary first premolars and reported that two-canal configurations corresponding to types II through VII were predominant in 89.64% of the observed cases, while type I with a single canal and types VIII or IX with three canals were observed in 8.66% and 1.66% of the cases, respectively (6). Similarly, in the second premolars, 50.64% of the cases were types II to VII, 48.66% were type I, and 0.66% had a three-canal configuration, corroborating the findings by Vertucci that only 1% of the maxillary second premolars had three canals (7).

As reported in the literature, maxillary premolars with three distinct roots—often called “ridiculous molars”—typically exhibit a mesiobuccal, distobuccal, and palatal root, each housing an individual canal. These are considered rare anatomical variations (8). This case report describes three clinical cases involving different three-rooted maxillary first and second premolars, highlighting the diagnostic approaches and clinical management strategies employed.

## 2 Clinical procedures

### 2.1 History, clinical examination, and general procedures

Three patients with varying chief complaints were referred to the postgraduate endodontic clinic at Ajman University, United Arab Emirates, for treatment. Their medical histories were reviewed and considered non-contributory in all cases. Intraoral examination revealed either large crown fillings or visible caries lesions. All necessary clinical and radiographic assessments were conducted, including periodontal probing, cold tests, and periapical radiographs, followed by preoperative cone beam computed tomography (CBCT) after identifying an additional root on radiography. Following the pulpal and apical diagnoses, all three cases were classified as highly difficult according to the case difficulty assessment of the American Association of Endodontists, and root canal treatment was recommended. Informed consent was obtained from all patients. Infiltration anesthesia (1.8 mL of 2% lidocaine with 1:80,000 epinephrine) was administered. Under rubber dam isolation, access cavities were prepared using round and Endo-Z endodontic burs. The pulp floor morphology was inspected under 25× magnification using a Leica M320 dental operating microscope (Leica Microsystems, Wetzlar, Germany). Orifices of the canals were refined with ultrasonic tips (ET 20D- Suprasson® P5 Newtron SATELEC; ACTEON, Merignac, France). Root canals were negotiated, and patency was achieved using stainless-steel ISO size 8–10K hand files (Dentsply Sirona, Ballaigues, Switzerland). Working length was determined with an electronic apex locator (Root ZX, Morita, Japan) and confirmed with periapical radiographs. Chemo-mechanical preparation of all canals was performed with different rotary systems, complemented by copious irrigation with 2% sodium hypochlorite (NaOCl) delivered using a side-vented 27G needle (Monojet, Covidien, Mansfield, USA). The final irrigation protocol consisted of NaOCl, followed by 17% ethylenediaminetetraacetic acid and then NaOCl, which was activated using Acteon® Irri Safe™ Passive Ultrasonic Irrigation files. The canals were dried with paper points and obturated with calibrated gutta-percha and resin-based sealer (AH Plus, Dentsply Sirona, Konstanz, Germany) using the continuous wave of condensation technique. Finally, pulp chambers were cleaned with alcohol, canal orifices sealed with flowable resin (3M Filtek Supreme Flowable Restorative, USA), and access cavities restored with composite restorations.

### 2.2 Case 1

**Figure 1:**
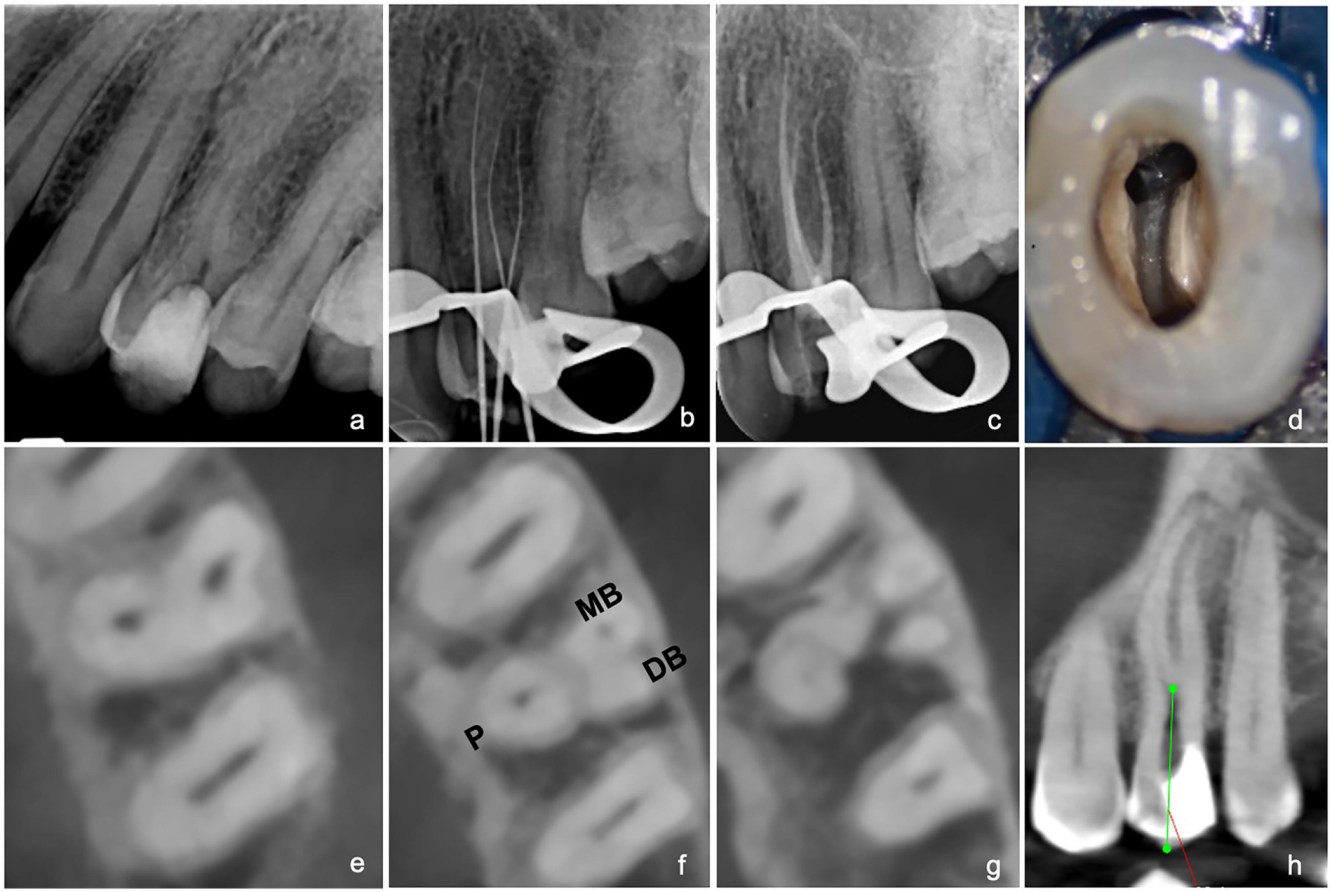
**(a)** Preoperative periapical radiograph of tooth #24. **(b)** Radiographic working length determination. **(c)** Postoperative radiograph confirming obturation. **(d)** Clinical view of pulp chamber showing buccal canal orifices. **(e)** Coronal CBCT view showing buccal and palatal canals. **(f)** Axial CBCT view at midroot level showing buccal canal bifurcation. **(g)** Axial view showing three separate roots at midroot level. **(h)** Sagittal CBCT view showing bifurcation of buccal canal at 12.4 mm.

A 28-year-old Sudanese man was referred by a general dentist for root canal treatment of the left maxillary first premolar (#24). The referring practitioner had initiated endodontic therapy but was unable to locate all canals due to the complexity of the root canal system. On examination, the tooth had no response to the cold test and no tenderness to percussion. The preoperative periapical radiograph did not clearly delineate the external root morphology (Figure 1a), so a CBCT scan of the tooth was requested. The scan revealed complex root canal anatomy with a long buccal canal bifurcation and three separate roots, with no visible periapical radiolucency. A diagnosis of previously initiated endodontic therapy with normal periapical tissue was established. The access cavity was refined utilizing an Endo-Z bur and ultrasonic tips to enlarge the buccal canal orifice and selectively remove dentin up to a depth of about 12.8 mm. This modification facilitated the exposure of the bifurcated canals and the removal of dentinal overhangs, thereby establishing straight-line access to both canals. Working lengths of the canals were determined and verified radiographically at 26 mm for the mesiobuccal (MB), distobuccal (DB), and palatal (P) canals. The Hyflex-CM rotary system (Hyflex CM, Coltene/Whaledent, Altstätten, Switzerland) was used for mechanical instrumentation. After all canals were shaped and disinfected, they were obturated as described previously (Figures 1a–h).

### 2.3 Case 2

**Figure 2:**
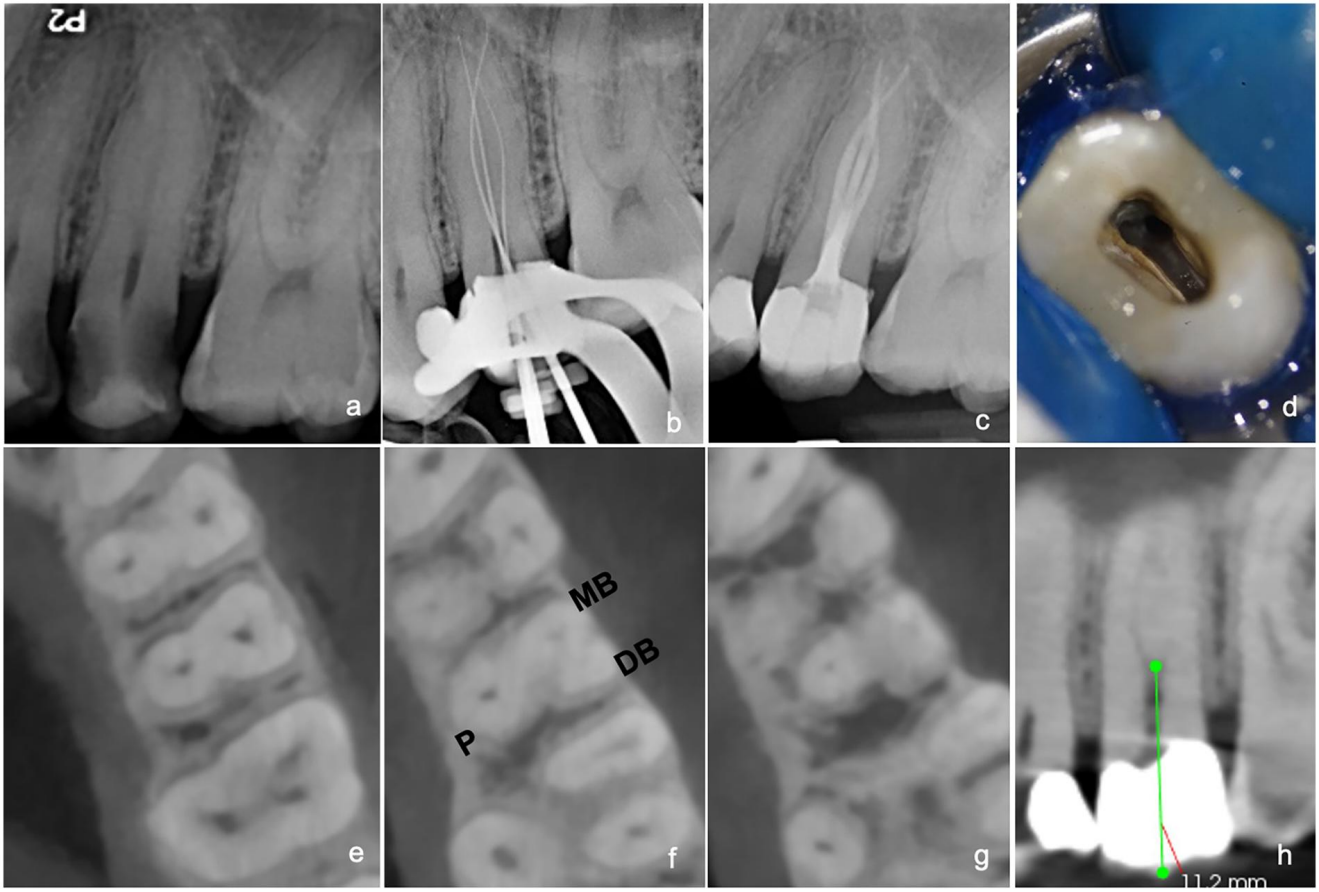
**(a)** Preoperative periapical radiograph of tooth #25. **(b)** Radiographic working length determination. **(c)** Postoperative radiograph. **(d)** Clinical image showing two buccal canal orifices. **(e)** Coronal CBCT view showing buccal and palatal canals. **(f)** Axial CBCT view at midroot level showing buccal canal bifurcation and three distinct roots. **(g)** Axial view showing apical confluence the confluence of buccal canals. **(h)** Sagittal CBCT view showing buccal canal bifurcation at midroot level.

A 22-year-old Sudanese man was referred for root canal treatment of the left maxillary second premolar (#25). The patient's primary complaint was severe, lingering pain on exposure to cold water. Cold sensitivity testing confirmed prolonged pain lasting over 30 s. The preoperative periapical radiograph revealed radiolucent areas on both the mesial and distal aspects of the tooth, suggestive of carious lesions. There was evidence of widening of the periodontal ligament space with no periapical changes (Figure 2a). Similarly, the preoperative periapical radiograph of tooth #25 could not clearly delineate the root morphology. Therefore, a CBCT scan was requested for a more detailed evaluation. Upon reviewing the CBCT, a canal bifurcation was observed in the coronal portion of the root at approximately 11.2 mm from the coronal reference point, resulting in two distinct canals, MB and DB, each with a separate canal path that then fused together along the length of the root at 17 mm and ended in one exit. Based on clinical and radiographic findings, a diagnosis of symptomatic irreversible pulpitis with normal periapical tissues was confirmed, and root canal treatment was initiated. Following the removal of the caries and the underlying weakened dentin, the access cavity was modified to facilitate a straight-line path into the canals. The working length of the canals was confirmed at 22 mm for the MB and DB canals and 23 mm for the palatal canal. The ProTaper Ultimate system (Dentsply Sirona, Ballaigues, Switzerland) was used for shaping the root canals. All other procedures were completed as described earlier (Figure 2).

### 2.4 Case 3

**Figure 3:**
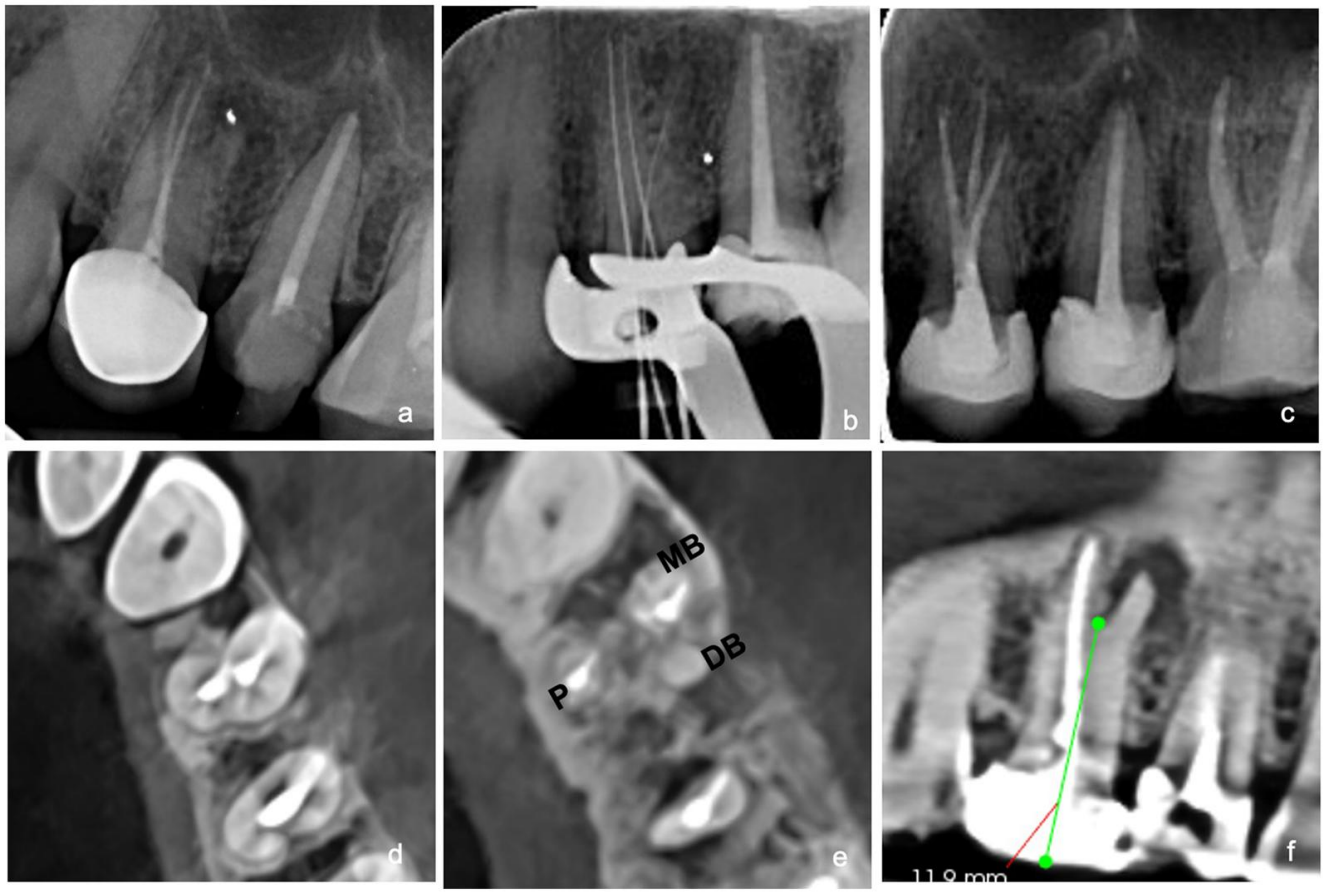
**(a)** Preoperative periapical radiograph of tooth #24. **(b)** Radiographic working length determination. **(c)** Postoperative radiograph. **(d)** Axial CBCT at cemento-enamel junction level showing buccal and palatal canals previously filled with gutta-percha. **(e)** Axial CBCT view showing three distinct roots and the missed distobuccal canal. **(f)** Sagittal CBCT view showing apical root bifurcation at approximately 11.9 mm from the coronal reference point.

A 42-year-old Sudanese woman presented to the postgraduate endodontic clinic with the chief complaint of pain and tenderness in the region of tooth #24. Clinical and radiographic evaluation confirmed a first premolar that had undergone previous root canal treatment and was restored with a crown. The tooth was tender to apical percussion, raising suspicion of a missed canal (Figure 3a). Therefore, a CBCT scan was performed for detailed evaluation. From the scan, a buccal canal bifurcation was observed in the apical portion of the root, resulting in two distinct canals—the MB and the missed DB—each with independent trajectories along the length of the root and associated with a large periapical lesion (Figures 3a–f). A diagnosis of a previously treated tooth with symptomatic apical periodontitis was established, with the missed canal identified as the most likely cause of the failure. After administering anesthesia, crown removal was initiated by sectioning the porcelain with a straight diamond bur, followed by cutting the metal part with a transmetal bur. Crown removal was completed with plastic instrument luxation. Removal of the previous obturation material was performed using Endo Star retreatment files (Poldent, UK). The working lengths were established at 17 mm for the palatal canal, 16 mm for the MB canal, and 15 mm for the newly identified DB canal. Canal preparation was performed using the WaveOne Gold Primary (WOG, Dentsply Maillefer, Ballaigues, Switzerland) file system. All canals were chemo-mechanically prepared and obturated as described earlier.

## 3 Discussion

Anatomical variations in root and root canal morphology of human teeth are not uncommon, and failure to identify them during treatment can greatly influence the treatment outcome (9). As reported, premolars have received considerable research attention, with overall prevalence of multiple canal morphology of 93.5% in first premolars and 49.7% in second premolars (6, 9). A three-rooted configuration has been observed in 1.8% of first premolars and 0.4% of second premolars. Asian populations generally show fewer roots and root canals, while European and African populations are more likely to present with higher frequencies of these anatomical features (9). A study of Sudanese adults found that 1.25% of maxillary first premolars had three root canals. In particular, one out of 80 maxillary first premolars examined exhibited a three-canal configuration. In contrast, 88.75% had two canals and 10% had a single canal (10).

Root canal treatment of maxillary premolars can pose significant challenges due to their complex and variable anatomy. These teeth often present with multiple roots, differing numbers of canals, varying pulp chamber configurations with longitudinal or directional depressions on the root surfaces, making it difficult to accurately interpret the apical anatomy on radiographs (11).

A comprehensive understanding of root canal anatomy, combined with precise diagnostic imaging, is essential before initiating root canal treatment (12). Periapical radiographs, offering only a two-dimensional view of a three-dimensional structure, are often insufficient in complex cases; hence, CBCT imaging is recommended as it offers higher resolution and improved geometric accuracy in assessing anatomical variations in the root canal system, ultimately contributing to better treatment outcomes and improved prognosis (13, 14). Similarly, the introduction of loupes and dental operating microscopes has significantly improved the visibility of the pulp chamber floor and root canal system. The American Association of Endodontists has emphasized the benefits of magnification for locating hidden canals, identifying cracks or fracture lines, removing canal obstructions, refining access preparations, and performing all aspects of endodontic microsurgery (15). In this case series, the use of CBCT and a dental operating microscope (Leica M320) for magnification and illumination enhanced the ability to explore the location of the additional canals and precisely modify the access cavity preparation.

Bilateral symmetry of anatomical variations has been reported in 64% of maxillary first premolars (16). In Case 1, the presence of three canals in the contralateral tooth was observed on the CBCT. This finding on CBCT highlights the importance of considering potential bilateral symmetry in root canal morphology variations when planning endodontic treatment on the opposite arch (17).

Clinically, the shape of the access cavity should be determined by the position of the canal orifices. In the maxillary first and second premolars, an oval-shaped access is typically recommended. However, when a third canal is suspected, identifying the closely positioned buccal orifices can be challenging. Therefore, modifying the access by extending a cut from the buccal canal openings to the buccal-proximal angle and creating a T-shaped access cavity design improves the access and visibility (18). This access cavity modification was applied in all cases to examine the pulp floor anatomy by carefully removing the dentin shoulder around the distobuccal canal orifice to achieve a straight-line access for effective cleaning and shaping, thereby minimizing the risk of missing additional canals (19).

The management of these cases relied on thorough clinical and radiographic assessment. At present, there is wide adoption of CBCT as part of routine radiographic investigation, and its use was critical in the cases presented here. However, its routine use should be clinically justified to avoid unnecessary radiation exposure. A limitation of this case series is the small sample size of three patients, all of Sudanese origin and treated at a single institution, which limits generalizability and applicability to other populations. In addition, the absence of long-term follow-up precludes assessment of sustained treatment outcomes. Future research should explore prospective or retrospective cohort studies with larger, representative sample sizes encompassing diverse ethnic populations to account for the documented variations in root canal anatomy across different demographic groups. Furthermore, longitudinal studies with extended follow-up periods are warranted to assess long-term clinical and radiographic outcomes.

## 4 Conclusion

This case series highlights that successful management of three-rooted maxillary premolars requires a systematic approach integrating thorough clinical examination, advanced radiographic imaging, and magnification. Clinicians should maintain a high index of suspicion for additional roots or canals, particularly when periapical radiographs suggest complex anatomy.

## Data Availability

The original contributions presented in the study are included in the article/Supplementary Material, further inquiries can be directed to the corresponding author.
